# Encapsulation of Catechin into β-Glucan Matrix Using Wet Milling and Ultrasonication as a Coupled Approach: Characterization and Bioactivity Retention

**DOI:** 10.3390/foods11101493

**Published:** 2022-05-20

**Authors:** Asima Shah, ZanoorUl Ashraf, Asir Gani, Faiza Jhan, Adil Gani, Munazah Sidiq

**Affiliations:** Department of Food Science and Technology, University of Kashmir, Srinagar 190006, India; zanoorashraf640@gmail.com (Z.A.); asir.gani@gmail.com (A.G.); fozia444@gmai.com (F.J.); adilgani@uok.edu.in (A.G.); wanimunazah94@gmail.com (M.S.)

**Keywords:** catechin, in vitro release behaviour, bioactivity retention

## Abstract

In this study, the nanoencapsulation of catechin into the β-glucan matrix from oats [O-Glu (C)] and barley [B-Glu (C)] was performed using the coupled approach of ultrasonication and wet milling. The nanoencapsulated catechin was characterised by particle size distribution, surface charge, SEM, and FTIR. The particle size was found to be 200 nm and 500 nm while zeta potential was found −27.13 and −24 mV for O-Glu (C) and B-Glu (C), respectively. The encapsulation efficiency of O-Glu (C) and B-Glu (C) was found to be 86.5% and 88.2%. FTIR and SEM revealed successful entrapment of catechin in β-glucan. The encapsulated capsules showed sustainable release during simulated gastrointestinal conditions. Moreover, both O-Glu (C) and B-Glu (C) showed that biological activity such as lipase inhibition activity and antioxidant assay were retained after in vitro digestion. It was concluded that O-Glu (C) and B-Glu (C) can be used as functional ingredients effectively in food as well as in the pharmaceutical field.

## 1. Introduction

Catechins are a group of polyphenolic compounds belonging to the class of flavonoids. They are active constituents present in tea, berries, cocoa, wines, etc., and possess a number of nutraceutical properties such as antioxidant, anticancer, anti-obesity, and antidiabetic, making them excellent for use in pharmaceutical and functional food industries [1,2,3,4]. Despite their abundant nutraceutical potential, catechins have some limitations, such as poor stability and absorption in the gastrointestinal tract, low solubility, reduced pharmacokinetics, scarce bio-distribution, and low targeting efficacy. Furthermore, oxygen and light are the utmost deleterious constituents of catechins [5,6,7]. To overcome these limitations, food scientists have used approaches such as encapsulation systems using different wall materials that include holding the biologically active compound in the matrix of a polymer to protect and deliver it safely to the targeted site, thereby improving its bioavailability [8,9]. For this purpose, nanoencapsulation is an innovative approach for protecting the catechin from deterioration owing to its increased surface-to-volume ratio for enhanced intracellular diffusion, surface activity, and increased bioaccessibility at the targeted site [10,11]. Different food-grade polysaccharide nanoparticles, which are biodegradable, biocompatible, and have improved functionality, show a good perspective for the protective delivery of bioactive compounds through human GI transit [12]. In recent years, different polysaccharide-based delivery systems, such as chitosan, starch, β-glucan, cellulose, etc., have been extensively used for nanoencapsulation [13,14,15]. Thus, for the present study, β-glucan from oat and barley has been selected for encapsulating catechin, as β-glucan is a functional ingredient that possesses different nutraceutical properties. Owing to these perspectives, this study was undertaken to nanoreduce β-glucan using wet milling technology followed by nanoencapsulation using ultrasonication.

Wet milling is a novel top-down technique that has been reported to be a more appropriate and promising method for fabricating compounds with low water solubility, and is also regarded as a green technology [16]. It involves the reduction in the size of particles into the nanometre range, thereby increasing surface areas which further enhance their physical properties [17]. This was followed by nanoencapsulation using the ultrasonication technique. Ultrasonication is also a physical green technique used for encapsulating the polyphenols of choice. It is relatively simple, fast, and produces a good yield [18]. It involves the use of sound waves of frequency >16 kHz to produce periodical waves that propagate through the medium. This produces bubbles that further collapse, creating high pressure and breaking the β-glucan matrix. It enables the entry of bioactive compounds within the polymer chain [19].

The main purpose of the current study was the nanoencapsulation of catechin into the β-glucan matrix and the evaluation of its effect on the release behaviour and bioactivity retention. It was hypothesised that this work would facilitate the use of a coupled approach which is a cost-effective, eco-friendly, and green technology to nanoencapsulate the sensitive bioactive constituents using β-glucan as a novel effective delivery system. Moreover, the developed capsules can be used as an ingredient in the development of the functional food with enhanced nutraceutical potential.

## 2. Materials and Methods

### 2.1. Materials

Oats (*Avena sativa*) and barley (*Hordeum vulgare*) were procured from a local market in Ladakh. The enzymes [α-amylase (*Aspergillus orygea* 30 U/mg), pepsin (porcine gastric mucosa 250 U/mg), bile salts, and pancreatin (porcine pancreas 8× USP),] and all the chemicals used for experimentation were procured from Sigma-Aldrich (St. Louis, MO, USA).

### 2.2. β-Glucan Extraction

β-glucan was extracted from oats and barley following the method of Ashraf et al. [20]. Barley and oat flour were dissolved in alkaline solution, heated for 40 min at 85 °C followed by centrifugation for 15 min, the supernatant was collected, its pH was adjusted to 4.5, and it was recentrifuged again. The recovered supernatant was treated with ethanol (99.9%) to precipitate β-glucan. Ethanol was air-dried, followed by enzymatic treatment, and the purity of β-glucan was found to be 92%, as evaluated using the megazyme β-Glucan Assay Kit.

### 2.3. Preparation of Nanoparticles

Oat and barley β-glucan solution was placed in the milling chamber of Dyno-mill Research Lab (WAB, Muttenz, Switzerland) equipped with the chiller system and milling beads. The speed of the milling was kept at 2500 rpm, with a milling power of 0.13 kW for 2 h. The temperature of the media mill was kept below 30 °C.

### 2.4. Nanoencapsulation of Catechin

Nanoencapsulation of catechin in the β-glucan matrix was performed following the method of Shah et al. [18]. The β-glucan sample (0.5 g) obtained from media milling was dissolved in 2% NaOH and heated (40 °C) until a glutinous solution was formed. The solution was then cooled and catechin (5 mg/mL) dissolved in ethanol was added drop wise into the β-glucan solution followed by ultrasonication at 40 kHz for 15 min with 5 s “onset” and 3 s “offset” at the temperature of 28 °C. The solution was then freeze-dried.

### 2.5. Characterisation of Nanoparticles

#### 2.5.1. Particle Size, Polydispersity Index and Zeta Potential

Dynamic light scattering (DLS) (Litesizer, Anton Paar, Ashland, VA, USA) was used to evaluate particle characteristics. The sample was prepared in distilled water at the concentration of 1 mg/mL and sonicated using a sonication bath (model: LTUSB) at 20 kHz for 15 min.

#### 2.5.2. Fourier Transform Infrared (ATR-FTIR) Spectroscopy

An ATR-FTIR Spectrophotometer (CARY 630, Agilent Technologies, Santa Clara, CA, USA) was used to evaluate the structural changes in B-Glu (C) and O-Glu (C) at the resolution of 4 cm^−1^ and within a range of 4000–500 cm^−1^.

#### 2.5.3. Scanning Electron Microscopy (SEM)

The morphology of B-Glu (C) and O-Glu (C) was visualised under the scanning electron microscope (Hitachi S-300H-Tokyo, Tokyo, Japan). The sample was placed on a gold-coated aluminium specimen stub and visualised under vacuum conditions.

### 2.6. Encapsulation Efficiency

Encapsulation efficiency was assessed following the method of Ashraf et al. [21] with some modifications. Briefly, 50 mg samples of B-Glu (C) and O-Glu (C) was washed with 5 mL of deionised water followed by centrifugation to remove the catechin stuck to the polymer surface. The supernatant was discarded and 50 mL of distilled water was added to the pellet, and the whole mixture was incubated for 24 h with continuous stirring at 37 °C. The mixture was centrifuged for 10 min and the absorbance of the supernatant was measured at 290 nm using a UV spectrophotometer. The catechin content in the supernatant was determined from the calibration curve

Y = 0.113X − 0.719 (R^2^ = 0.9968)

where, Y is the absorbance of catechin and X is theconcentration of catechin in mg/mL.

The encapsulation efficiency was determined using the formula:

$$\mathrm{Encapsulation}\;\mathrm{efficiency}\;(\%)=\frac{\mathrm{Quantity}\;\mathrm{of}\;\mathrm{catechin}\;\mathrm{loaded}\;\mathrm{in}\;\mathrm{the}\;\mathrm{powder}\;}{\mathrm{Quantity}\;\mathrm{of}\;\mathrm{catechin}\;\mathrm{added}}\times 100$$

### 2.7. Swelling Behaviour

The swelling behaviour of the samples was evaluated at different pH values of 3, 4 and 7.5. following the method of Shah et al. [19]. Sample (M_1_) was dissolved in phosphate-buffered saline (10 mL) maintained at different pH values of 3, 4 and 7.5 and incubated for 2 h at 37 °C. This was followed by centrifugation (1000× *g*) for 10 min. The supernatant was discarded and the pellet was weighed (M_2_). The gain in weight was calculated using the formula

$$\mathrm{Swelling}\;\mathrm{behaviour}=\frac{\;\left(M2-M1\right)}{M1}\;\times \;100$$

### 2.8. In Vitro Release Behaviour of Catechin

A simulated gastrointestinal model was used to evaluate the release behaviour of catechin following the method of Gani et al. [13] with some modification. Briefly, 100 mg of encapsulated nanocapsule was dissolved in α-amylase solution (0.2%, pH 7.2) (prepared in phosphate-buffered saline) in order to simulate the mouth environment for 5 min. The sample was then centrifuged (5000× *g*) for 10 min and the supernatant was read at 290 nm using a spectrophotometer to determine the release of catechin. This was followed by exposing the pellet to gastric digestion in intervals of 30 and 60 min using pepsin (3 g/L prepared in NaCl (9 g/L) pH 3). The sample solution was recentrifuged and the release of catechin from the β-glucan matrix was evaluated by checking the absorbance of the supernatant at 290 nm. The recovered pellet was again subjected to simulated intestinal juice using bile salts (3 g/L) and pancreatic enzyme (10 g/L) prepared in phosphate-buffered saline (pH 7.5) and then incubated at 37 °C with mild stirring followed by centrifugation at the interval of 30, 60 and 120 min. Absorbance of the supernatant was again observed to evaluate the catechin released. The amount of catechin released was evaluated using the equation in Section 2.6.

### 2.9. Retention of Antioxidant Activity after Gastrointestinal Digestion

B-Glu (C) and O-Glu (C) was exposed to simulated human digestive environment and their biological activities, such as anti-obesity and antioxidant activity was evaluated using in vitro antioxidant and anti-obesity assays. Sample (50 mg) was dissolved in simulated gastrointestinal juice following the method of Gani et al. [13] and the supernatant was collected for biological activity evaluation.

#### 2.9.1. In Vitro Anti-Obesity Activity

##### Pancreatic Lipase Inhibition Activity

The in vitro anti-obesity activity was assessed following the procedure of Shah et al. [18]. Briefly, a solution mixture consisting of 50 μL sample solution, 25 μL of enzyme solution (50 mg/mL) and 25 μL p-Nitrophenyl butyrate (10 mM prepared in acetonitrile) prepared in sodium phosphate buffer (0.2 M, pH 7.2) was incubated at 37 °C for30 min. The absorbance (AB) was measured at 405 nm using a spectrophotometer and the inhibition activity was evaluated using Equation (1).

% Inhibition = 1 − [(P − S)/(Q − R)](1)

where Q is the absorbance of the control without test sample, R is theabsorbance of a control blank without a test sample and enzyme, P is the absorbance of the reaction mixture, and S is theabsorbance of a reaction blank with the test sample but without the enzyme.

##### Cholesterol Esterase (CE) Inhibition Assay

A reaction mixture containing the sample (50 µL), *p*-nitrophenol butyrate pNPB (25 µL), and a 50 µL solution of CE (10 µg/mL) was incubated for 30 min at 37 °C. After incubation, the release of p-nitrophenol was observed at 405 nm, and the percent enzyme inhibition was determined following Equation (1).

#### 2.9.2. In Vitro Antioxidant Assay

##### DPPH Activity

Radical scavenging activity was estimated as described by Gani et al. [22]. The solution mixture contained the sample (100 μL), DPPH solution prepared in methanol (100 μL) and 800 μL of methanol to make up the volume of 1 mL. The whole mixture was incubated for 40 min in the dark at a temperature of 20 °C and absorbance of the sample was read at 517 nm.

Percentage inhibition was calculated by Equation (2):

(2) $$\mathrm{inhibition}\;\left(\%\right)=\frac{p-a}{p}\times 100$$

where (p) is the absorbance of the control and (a) is the absorbance of the sample.

##### Reducing Power

The reducing power activity was assessed following the method of Gani et al. [23]. A reaction mixture containing 1% potassium ferricyanide, 2.5 mL sodium phosphate buffer (0.2 M), and 2 mL sample was incubated for 20 min at 40 °C. This was followed by terminating the reaction using TCA (10%), and then by centrifuging the whole reaction mixture for 10 min at 3000 rpm. The supernatant was then diluted with distilled water and the absorbance of the sample was observed at 700 nm. The percent reduction was calculated by Equation (3):

(3) $$\mathrm{Reduction}\;(\%)=1-\left[1-\frac{\mathrm{Ac}}{\mathrm{As}}\right]\times 100$$

where (Ac) is the absorbance of the control and (As) is the absorbance of the sample.

### 2.10. Statistical Analysis

All the experiments were carried out in triplicates and the data were presented as mean ± standard deviation. One way analysis of variance (ANOVA) with Duncan’s test and paired T-sample test was used to analyze the results using statistical software (IBMSPSS statistics 21). 

## 3. Results and Discussions

### 3.1. Particle Size, Polydispersity Index, and Zeta Potential

The distribution of particles in solution is a significant characteristic feature to know the physicochemical behaviour of the encapsulant. It affects the solubility, release rate, and encapsulation efficiency of the particles. The average particle size distribution of wet milled B-Glu and O-Glu was found to be 130.1 and 313.2 nm. Wet milling uses mechanical energy to break down the large particles into fine powder, thereby enhancing its stability, bioavailability, and functionality [24]. However, after encapsulation, the average diameter of particles was found to be 200 nm and 498 nm for B-Glu (C) and O-Glu (C). An increase in particle size upon nanoencapsulation might be due to the incorporation of the catechin molecule inside the β-glucan matrix or it may be attributed to the interactions between the catechin molecule and the β-glucan matrix. Ahmad et al. [25] reported similar size distribution of catechin encapsulated in starch extracted from different sources.

The polydispersity index (PI) determines the heterogeneousity of the sample on the basis of the size, which affects the dissolution and accessibility/reactivity of the biopolymer [26]. As per the ISO (International Organization for Standardization), PI values less than 0.05 are monodisperse in nature, whereas PI values of more than 0.7 are polydisperse in nature [27]. The PI values for B-Glu and O-Glu was found to be 0.1 and 0.2; however, for B-Glu (C) and O-Glu (C) it was found to be 0.2 and 0.4, indicating that both wall materials (B-Glu and O-Glu) and B-Glu (C) and O-Glu (C) are monodisperse in nature, and hence have higher uniformity and stability. Zeta potential (ZP) depicts the charge of particle surface and gives knowledge about repulsive forces between particles. The higher the value of zeta potential, the higher the electrostatic repulsion will be, and the weaker the van der Waals forces between the particles will be, hence preventing particle agglomeration [26]. ZP influences the release kinetic and biological fate of nanoparticles [28]. As can be seen in Table 1, B-Glu and O-Glu had negative surface charges of −11 and −14 mV, whereas −24 mV and −27.1 mV for O-Glu (C) and B-Glu (C). The negative values of zeta potential demonstrate that the surface charge of the samples was negative.

### 3.2. Structural Elucidation Using FTIR

**Figure 1:**
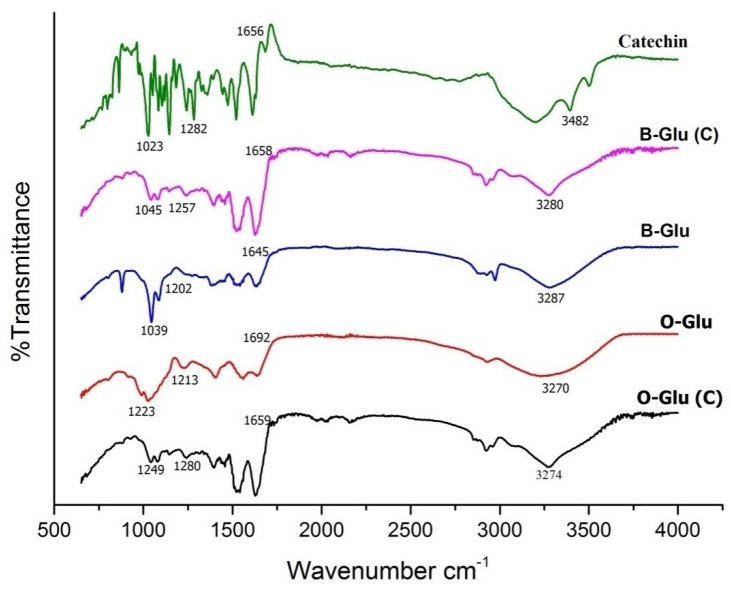
The FT-IR spectrum of catechin, B-Glu (C), B-Glu, O-Glu (C), O-Glu. B-Glu (C) represents catechin encapsulated in barley β-glucan, O-Glu (C) catechin encapsulated in oats β-glucan, B-Glu represents barley β-glucan, and O-Glu represents oats β–glucan.

The FTIR spectra of β-glucan, catechin and encapsulated capsules are shown in Figure 1. The spectrograph shows the appearance of many new bands shifting, broadening, and decreasing in the intensity of absorption peak. This depicts the probable interactions of catechin with β-glucan matrix. The infrared spectra of β-glucan from oats and barley showed a broad peak at a wavelength of 3000–3500 cm^−1^ corresponding to OH groups, 2922–3000 cm^−1^ corresponding to C–H stretch, 1654.55 cm^−1^ corresponding to bending vibrations of bound water, 989–1200 cm^−1^ corresponding to C=O and C-C vibrations, and a characteristic peak of β-glucan at 889 cm^−1^ [10,13,29]. The spectra of catechin displayed peaks at 3482 cm^−1^ representing O–H stretching regions, and 1656 cm^−1^, 1285 cm^−1^, 1150 cm^−1^ and 1071 cm^−1^ representing the existence of aromatic rings [25]. However, B-Glu (C) and O-Glu (C) exhibited representative peaks of catechin (in the range of 3274–3278 cm^−1^ 1645–1692 cm^−1^, 1213–1285 cm^−1^, and 1071 cm^−1^), indicating the successful encapsulation of catechin in β-glucan. Similar results were also obtained for catechin encapsulated in starch where the nanoparticles had decreased and reframed peaks [25].

### 3.3. Scanning Electron Microscopy

**Figure 2:**
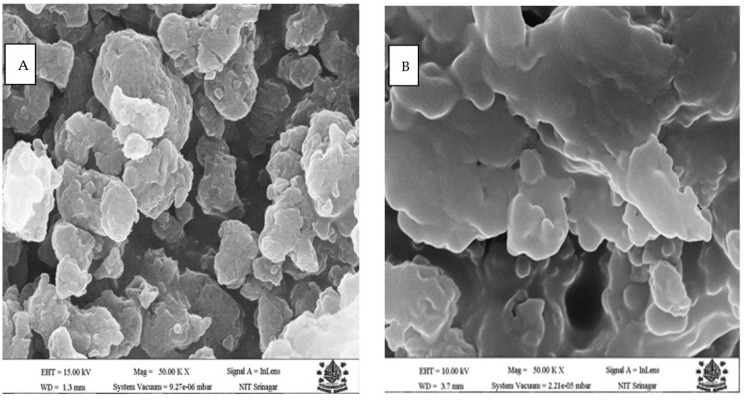
The SEM images of B-Glu (C) (**A**) and O-Glu (C) (**B**). Here, B-Glu (C) represents catechin encapsulated in barley β-glucan, O-Glu (C) catechin encapsulated in oats β-glucan

The Scanning electron microscopy evaluation of O-Glu (C) and B-Glu (C) showed different morphological characteristics, as presented in Figure 2. The B-Glu(C) micrograph displayed a spongy, porous structure with surface roughness. On the other hand, micrographs of O-Glu (C) displayed an angular/ellipsoid shape with a smooth surface. Both B-Glu (C) and O-Glu (C) displayed heterogeneity in the structure. Similar results were also described by Tarun et al. [30], in which drug-loaded glucan particles were found to be more heterogeneous and ellipsoidal in shape.

### 3.4. Swelling Behaviour

**Figure 3:**
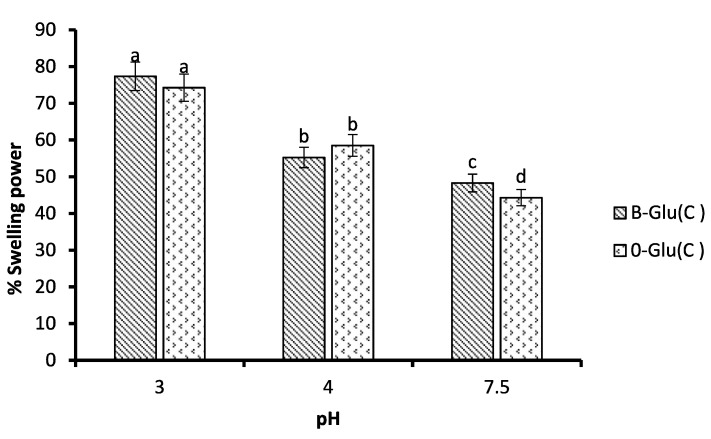
Swelling power of B-Glu (C) and O-Glu (C). B-Glu (C) represents catechin encapsulated in barley β-glucan, and O-Glu (C) is catechin encapsulated in oats β-glucan. Different letters on the bars at different pH indicate significant difference (*p* < 0.05).

The swelling behaviour involves the rehydration of B-Glu (C) and O-Glu (C) in phosphate buffer maintained at pH 3, 4, and 7.5 to simulate the gastric and intestinal environment. It can be inferred that if B-Glu (C) and O-Glu (C) swell without disintegration over a period of time reaching the peak, the particles have retained catechin and hence are more stable, and vice versa. The swelling index values of B-Glu (C) and O-Glu (C) wasfound to be 77.34% and 74.24% at pH 3 and 55.25% and 56.51% at pH 4, as shown in Figure 3. Results revealed both the simulated conditions for B-Glu (C) and O-Glu (C) varied (*p* ≤ 0.05) and revealed that catechin can be retained well in an acidic medium. Additionally, an increase in the swelling index may be because of the breaking up or unfolding of β-glucan linkages [13]. However, under alkaline conditions, the swelling index values were found to be 48.3% and 44% for B-Glu (C) and O-Glu (C), respectively, which might be due to the disintegration of nanocapsules leading to a decrease in the values of the swelling index and, in turn, increase in the release of catechin (Figure 3).

### 3.5. Encapsulation Efficiency (EE)

**Figure 4:**
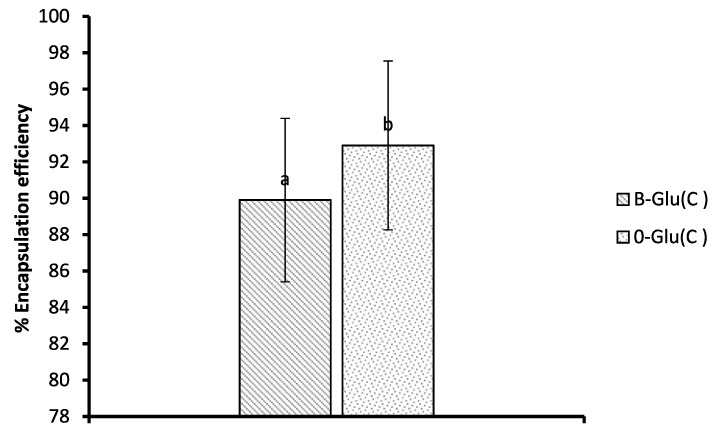
Encapsulation efficiency of B-Glu (C) and O-Glu (C). Here, B-Glu(C) represents catechin encapsulated into a β-glucan matrix and O-Glu(C) represents catechin encapsulated into oats β-glucan matrix. Different letters on the bars indicate significant difference (*p* < 0.05).

Encapsulation efficiency (EE) gives us knowledge about the quantity of the drug enclosed inside the core of the polymeric matrix. Various factors that affect the encapsulation efficiency are the size of the particle, type of wall material, and the method of encapsulation [15]. The loading capacities of B-Glu (C) and O-Glu (C) was found to be 3.32 and 4.09 mg/mL. The EEs of B-Glu (C) and O-Glu (C) was found to be 89.9% and 92.2%, as shown in Figure 4. The encapsulation efficiency for catechin-laden oats and barley β-glucan specifies significant variance in the amount of catechin holding ability. Good EE of B-Glu (C) was found in comparison withO-Glu (C), which may be attributed to the difference in the glycosidic linkage of the β-glucan which may be significantly influenced by the type and the source of wall material used, and hence having different retention and film-forming capacity [31]. Our results of EE are higher than the study reported by Ahmad et al. [25]. An increase in the EE of the β-glucan from oats and barley could be due to the narrow size of the particles. Narrow particle size means good EE because of the capability to form a good film around the core material, therefore allowing better retention of encapsulated molecules [32].

### 3.6. Release Behaviour of Catechin

**Figure 5:**
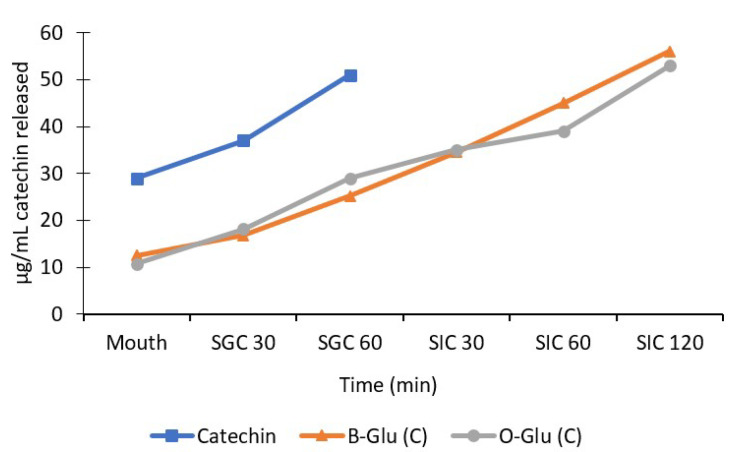
In vitro release pattern of catechin from the β-glucan matrix. SGC (simulated gastric condition) at 30 and 60 min of incubation; SIJ (simulated intestinal condition) at 30, 60, and 120 min of incubation.

The main aim of nanoencapsulation was to prevent the bioactive compound from harsh conditions of gastrointestinal tract and enable its release at the targeted site to retain the desired health benefits. The nanoencapsulated and free catechin was subjected to a simulated human digestion model where the release behaviour was studied, as depicted in Figure 5. The release of catechin from nanocapsules varied significantly under simulated gastric and intestinal conditions [13,18,20]. The amount of catechin released in the mouth was found to be 12.5 µg/mL for B-Glu (C), 10.7 µg/mL for O-Glu (C), and 29 µg/mL for free catechin. The quantity of free catechin released was significantly higher in comparison to encapsulated nanocapsules. However, the preliminary release of catechin from B-Glu (C) and O-Glu (C) in the mouth can be ascribed to catechin adhered to the β-glucan surface with weak bonds [13]. Furthermore, under simulated gastric conditions, the quantities of catechin released during the 30 min and 60 min varied significantly as the bioactive carrier system degraded gradually and the release of catechin increased significantly. The amount of catechin released was 16.8–25.2 µg/mL for B-Glu (C), 18.2–29 µg/mL for O-Glu (C), and 37–51 µg/mL for free catechin. The free catechin was fully released under gastric conditions. An interpretation can be made from the result that pH is lower in the stomach; hence, the swelling ratio is higher [33]. A similar release trend was observed by Gani et al. [13], Shah et al. [18] and Chin et al. [34] for the release of vitamin D3, rutin, and curcumin from carbohydrate-based nanoparticles, respectively. Furthermore, it was observed that the release pattern of catechin under Simulated intestinal conditions after 30, 60 and 120 min varied in the range of 34.5–56 µg/mL for B-Glu (C) and 35–53 µg/mL for O-Glu (C). Moreover, the bile digestion for 120 min showed an increased release of catechin under the intestinal condition which is concerned with the decreased swelling of the capsules and higher pH. This resulted in the disintegration of nanocapsules due to the increased surface area, and hence, increased release of catechin. The results are in coordination with the swelling power (Figure 3). A similar release pattern of vitamin D3, rutin, and α-tocopherol from β-glucan nanoparticles was observed previously [13,18,21].

### 3.7. Retention of Biological Activity of Catechin after Digestion

The biological activity of B-Glu(C) and O-Glu(C) was studied before and after simulated gastrointestinal digestion (SGID) conditions. Commonly, the biological activity of bioactive compounds is assessed directly; however, the assessment of biological activity once swallowed into the human body would be more interesting to know. Furthermore, encapsulation into a wall material is expected to augment the biological activity of encapsulant by securing its passage to the lower GI tract. The biological activity of encapsulated catechin was observed in terms of antioxidant and anti-obesity activities.

#### 3.7.1. In Vitro Antiobesity Activity

**Figure 6:**
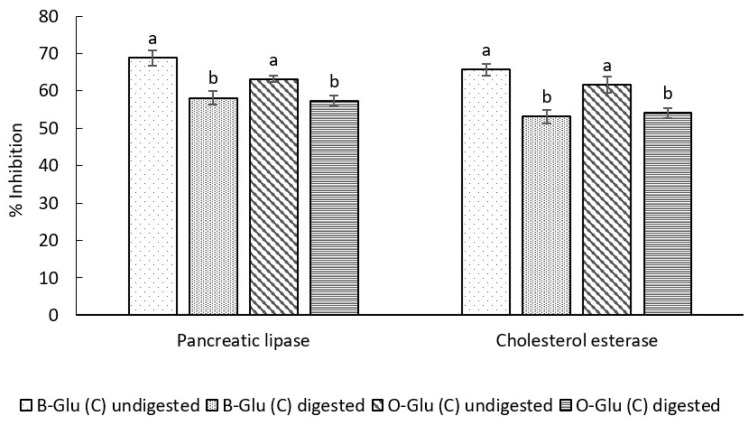
Anti-obesity activity of B-Glu (C) and O-Glu (C). Here, B-Glu (C) represents catechin encapsulated into barley and O-Glu(C) represents catechin encapsulated into oats β-glucan matrix. Different letters on the bars representing same sample before and after digestion indicate significant difference (*p* < 0.05).

The in vitro antiobesity activity was evaluated by assessing the percentage inhibition of pancreatic lipase (PL) and cholesterol esterase enzyme (CE) activity (Figure 6). Before SGID the PL and CE activity was found to be 68.7% and 65.6% for B-Glu (C) and 63.2% and 61.7% for O-Glu (C). After digestion, reduction of 15% in PL and 19% in CE activity was observed in B-Glu (C) and 9% in PL and 12% in CE activity was observed in O-Glu (C) (*p* < 0.05). Results indicated that the release of catechin from the β-glucan matrix was not considerably affected by pH during SGID. Encapsulation of catechin in the β-glucan matrix aimed to protect the catechin from harsh gastrointestinal conditions until it is released and absorbed at the targeted site and exerts its valuable health benefits. Our results are in accordance with studies in the literature, which represent the similar release behaviour of bioactive compounds under SGID [18]. Similar results of bioactivity were reported for catechin encapsulated in starch nanoparticles by Ahmed et al. [25].

#### 3.7.2. Antioxidant Activity 

**Figure 7:**
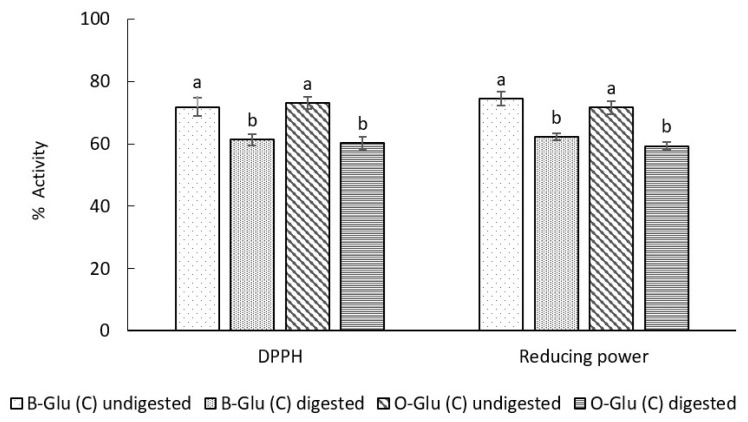
Antioxidant activity of B-Glu (C) and O-Glu (C). Here, B-Glu (C) represents catechin encapsulated into barley and O-Glu (C) represents catechin encapsulated into oats β-glucan matrix. Different letters on the bars representing same sample before and after digestion indicate significant difference (*p* < 0.05).

The antioxidant activity was evaluated before and after the digestion of encapsulated capsules in terms of the percentage inhibition of radical scavenging activity and reducing power (Figure 7). Before SGID the DPPH scavenging activity was found to be 71.8% for B-Glu (C) and 73.1% for O-Glu (C) and % reduction was found to be 74.5 for B-Glu (C) and 71.6 for O-Glu (C). After digestion, reduction of 15% in DPPH scavenging activity and 17% in reducing power was observed in B-Glu (C) and 18% in DPPH scavenging activity and 17% in reducing power was observed in O-Glu (C) (*p* < 0.05). The digest from B-Glu (C) exhibited higher antioxidant activity than that O-Glu (C), which might be due to the structural difference of the source of β-glucan, and different dispersion pattern [35]. Results indicated that the release of catechin from the β-glucan matrix was not considerably affected by pH during SGID. Additionally, increased surface area might have contributed to higher digestion of the β-glucan, and hence, the release of catechin from β-glucan is higher. Our results are in accordance with studies in the literature, which described the similar bioactivity of quercetin from polysaccharide nanoparticles [35]. Similar bioactivity retention of vitamin D_3_ encapsulated in β-glucan nanoparticles has been reported by Gani et al. [13].

## 4. Conclusions

This study concluded that the encapsulation of catechin using a coupled approach of wet milling and ultrasonication technique can overcome the problems of low solubility, stability and bioavailability into food systems using β-glucan as wall material. The B-Glu (C) and O-Glu (C) showed good encapsulation efficiency and controlled release behaviour with retained biological activity under simulated gastrointestinal conditions. It was concluded that developed nanocapsules can be used to fortify different food ingredients for the development of functional food. This study is expected to pave the way for nanoencapsulating different bioactive constituents. Further research needs to focus on the structural profile, toxicity, and functional properties of nanocapsules. The perspective of the catechin encapsulated into β-glucan as a functional ingredient in different food products also needs to be determined.

## Figures and Tables

**Table 1:** DLS results.

	B-Glu	B-Glu (C)	O-Glu	O-Glu (C)
Particle Size (nm)	130.1 ± 1.2 ^a^	200 ± 1.5 ^b^	313.2 ± 2.1 ^c^	498 ± 1.9 ^d^
Polydispersity index (PDI)	0.1 ± 00 ^a^	0.2 ± 00 ^a^	0.23 ± 00 ^a^	0.4 ± 00 ^a^
Zeta potential (mV)	−11 ± 1.1 ^a^	−27 ± 1.3 ^b^	−14 ± 1.1 ^a^	−24 ± 0.2 ^c^

Results are expressed as means ± S.D (*n* = 3). Values followed by same letter in a row do not differ significantly (*p* > 0.05). Here, B-Glu represents barley β-glucan, O-Glu represents oats β-glucan, B-Glu (C) represents catechin encapsulated into a β-glucan matrix, and O-Glu (C) represents catechin encapsulated into the oats β-glucan matrix.
